# An Inquiry into the Sources and Mode of Action of the Poison of Fever

**Published:** 1841-07-01

**Authors:** Alfred Hudson

**Affiliations:** Physician to the Navan Fever Hospital


The objection assumes that typhus confers no immunity from subsequent attacks and that the exanthemata do confer an immunity. The answer is, that experience warrants our belief in a considerable power of destroying the liability to subsequent attacks in typhus, and that, though there can no doubt of the exanthemata possessing this power, exceptions to it are frequent in all of them. It must be admitted that in this country there is a general belief in the protecting power of a seasoning or initiatory fever, and though we rarely meet with a medical man who has not had typhus, we certainly meet with few indeed who have had it more than once. The nature of the subject does not admit of very precise proof. We can only obtain the *general results* of experience. Dr. Barker* states as the results of his, "that he has for some time entertained the opinion that sufferers from fever attended with this eruption, if they are not altogether secured by it from a second attack, are not at least so liable to it as those who have had a fever of the ordinary kind; and, though he frequently made the enquiry, he never found a patient in whom this symptom was distinct who had suffered from the same fever on any former occasion." Dr. Perry† states as the result of an extensive series of observations his opinion, "that typhus generally is taken but once in a life-time, and that a second attack does not occur more frequently than of small-pox, and less frequently than of measles or scarlatina." Dr. Davidson states that of 609 patients in the Glasgow fever hospital only 74 stated that they had ever had fever previously. He justly observes that when we take into account the various diseases which are confounded with typhus, this small number can be easily accounted for.

But the protective power of the exanthemata has been much overrated. Three instances of second attacks of small-pox came to my knowledge in this county very recently. In two of them the patients had suffered the disease from inoculation very few years before. In one in which the inoculated disease was severe a most confluent eruption accompanied the second attack seven years after. Another instance has been related to me of a lady living in this country who has had the disease three times. Dr. Roupell refers to the case of one who had it seven times.

Instances of second attacks of measles are given by Dr. Bailie, who attended five children in May, and again in the following November, by Dr. Webster,‡ and by Rayer, who states that he met with three instances of second attacks of measles in the interval between the publication of the first and second editions of his work. The remarkable case of a second attack by Dr. Graves § should, perhaps, be termed relapse into measles. The second eruption appeared twenty-one days after the commencement of the first illness, in which the eruption had been copious and severe.

Cases of second attack of scarlatina are stated by Roupell to be not at all uncommon. Several have fallen under my own observation.

V. The liability to relapse in cases of typhus has been urged as an objection to the classification by Harty and others. It might be replied that cases of measles, such as that of Dr. Graves just referred to, and cases of *reversio*, as it is termed by Rayer, after scarlatina, would tend to shew that the exanthemata are not exempt from relapse. But the true answer is that typhus *is peculiarly exempt* from relapse. Two kinds of cases are erroneously considered such: 1st. Relapses from typhus into fever symptomatic of a visceral irritation—generally gastro enterite.—" I am persuaded, says Cheyne, || that obstinate and fatal

---

* Dublin Medical Transactions. Vol. 2.

† Dublin Med. Journal, Vol. 10, and Edin. Med. and Surg. Journal, Jan. 1836.

‡ Medico-Chirurgical Transactions. 2nd Series. Vol. 4.

§ Dublin Medical Journal, Nov. 1840.

|| Dublin Hospital Reports. Vol. 1.

relapses after typhoid fevers are often attributable to inflammation and, perhaps, ulceration of the villous coat of the intestines." And Broussais asserts that "when the frequency of pulse in 'convalescence' does not diminish, and the strength does not increase, it may be suspected that a form of latent inflammation exists. It may be discovered by permitting an excess which generally changes this frequency into a *real fever*, and developes the pain *of* the irritated part;"* but, 2ndly, cases of fevers not typhoid will under exposure to infection relapse into typhus. Dr. Davidson gives a tabular view of the relapses in the Glasgow Fever Hospital among 686 cases, in which no case of relapse from typhus into typhus occurred, but two of febricula and one of intestinal fever into typhus.

In 500 cases of fever admitted into the Navan Hospital in 1840, two cases only of relapse into typhus occurred, both were cases of febricula, which after a few days were sent to the convalescent ward, where they relapsed into maculated typhus, one in four days and the other in fourteen days after their removal thither.

VI. Lastly, the following extraordinary objection is put forward by Dr. West:—†

"The type of the fever itself varies, being sometimes intermittent, sometimes continued, changing from the one to the other form, and being occasionally converted into other diseases."

In other words there is no such disease as typhus!

To this the supporter of the speciality of typhus replies, that the disease is here, and in numerous quotations throughout the paper, confounded with other fevers; typhoid it may be in their nature, or becoming so in their progress, not arising from an animal infectious poison, but from a variety of sources, which contain a variety of poisons, the identity of *any one of which* with the *typhus* poison is a matter in dispute, and to be argued upon the conclusion of our investigation into these sources in the following chapter.

Meantime the pertinent remarks of Dr. Copland, upon this subject, are not unworthy the notice of those who rely for the means of drawing accurate distinctions upon such sources as Dr. West has explored:—" *True* or *contagious* typhus has been confounded with synochoid and nervous fevers on the one hand, and with putrid or malignant fevers on the other. It has been already stated that putridity or malignancy, not only may characterize a particular form of fever or certain epidemics even at an early period of their course, but, also, owing to various contingencies, may take place in advanced stages of any other fever. As the circumstances favouring the generation and spread of typhus are often such as also tend to develope those changes which have been usually named putrid or malignant, and as these changes are frequently observed in the latter stages of typhus—the symptoms distinguishing this fever becoming associated with, or followed by, those indicating the putro-adynamic state—so has it been often confounded with other fevers in which this state has predominated more or less. If we refer to the numerous histories of epidemic typhus, recorded by writers from the close of the 15th century up to the present time, we shall find that although many of these, owing to the concurrence of circumstances, developing a putrid or malignant disease, were instances of fever either identical with or very closely resembling that which I have described as such in the preceding section, yet many others, or even the majority, were true typhus, in which the putro-adynamic state was either early or predominantly developed. The exanthematous eruption, characteristic of typhus, being succeeded or accompanied by the petechiæ, indicating the approach of the septic condition, and being either

* Chronic Phlegmasiæ, Vol. 2, p. 53.

† On Exanthematous Typhus.

mistaken for them or for an eruption of miliaria. Owing to this circumstance, especially typhus, low, nervous, and putrid fevers, have been very generally confounded together."

The reader of the foregoing, and many other passages in Dr. Copland's admirable article on typhus, must be startled with the following passage in Dr. Roupell's recent work on the disease, when he finds that Dr. Copland is not like Peebles and others, who have described exanthematous typhus, passed over in silence, but is actually mentioned by name as belonging to the authors referred to.

"In the above description *typhus* is considered to belong to the continued fevers. It is looked upon by the most recent authors, in this and other countries, not as an individual disorder, but as one into which others may be and frequently are converted!" (page 5.)

Here for the present we leave the subject, since that portion of the argument for the classification of typhus with the exanthemata, which is derived from the differences between it and other continued fevers, will properly come under consideration when discussing the identity of typhus and typhoid fever.

### Chapter II.—Of a Fever Poison, generated during the Decomposition of Dead Organic Matters.

The difficulties attending an examination into this part of the enquiry into the sources of fever are very great, and are confessed by all who are familiar with the conflicting statements advanced on the subject. Our difficulties are increased by the indeterminate character of much of the evidence offered in proof of the paludal origin of fever, some of which not only claims to prove the power of such sources to cause continued, but *infectious* fever—by the fact that much of this evidence is moreover inadequate, as it proves only the occurrence of fever in situations and among persons which might be considered equally obnoxious to contagion as to miasm, and by the silence, or mysterious, or contradictory language of those to whom we might look for assistance and direction in a scrutiny of the mass of conflicting testimony, from which our conclusions are to be drawn.

Thus, Dr. Christison says, "the great questions involved in the investigations into the causes of continued fever are three in number:—Does the disease originate in infection? Does it originate in other causes? Granting that it does originate in other causes, may such fevers propagate themselves by infection? It will be seen that they cannot be all answered by any means with equal confidence;" and, accordingly, while he is full and illustrative on the subject of contagion, he treats of other causes in a most cursory and unsatisfactory manner, and while he admits that "the general conclusion from the whole facts seems to be that a disease, undistinguishable from true infectious fever, may sometimes arise without infection," adds, "that on descending from the general question to the more special one—what the other cause or causes of fever may be?—the difficulties are greatly increased, indeed they become insurmountable, without such limited and vague facts as are at present possessed on the subject," and "it appears a needless waste of time and labour to attempt anything further on this head."

Nor are we more enlightened by Dr. Davidson, who, while he states that he is not prepared to assert that febrile affections may not, under peculiar circumstances (what he does not inform us), arise from paludal sources, effectually excludes them from consideration by putting forward the following conclusions under the head of "Alleged sources of continued fevers, *not* typhoid."

"From a consideration of the whole evidence that might be adduced respecting this point, it may be drawn as a conclusion, that although putrid matters when injected into the veins of animals cause death under symptoms similar to those of typhus fever, yet that the effluvia arising from similar matters do not, under ordinary circumstances, produce any deleterious effects on man." Again—"Before concluding this part of the essay we shall notice an hypothesis, which has lately been somewhat confidently brought forward to account for the prevalence of typhus in some large cities, viz.: that a peculiar malaria is generated by the animal and vegetable filth, which accumulates along the sides of rivers running through large towns, and that the inhabitants who live in their immediate vicinity become thereby subject to fever. We are quite aware that very disagreeable and sometimes fœtid effluvia occasionally arise from such situations, particularly during hot weather, but that it is capable of causing continued fever has not even been rendered probable by any satisfactory evidence."

We have it stated upon high authority that gaseous contagions contain organic matter in a state of decomposition or progressive change. We have it also announced that from certain decomposing animal and vegetable substances, organic matter in a state of "progress to decay" is evolved, which, when collected and retained in a manner similar to the former, completes the stage of decomposition, or, in other words, "putrefies."

By evidence of the most unexceptionable kind the former of these is proved to be capable of communicating the *state of change*, of which it is the subject, to the healthy human organism.

We have to enquire whether the analogy of *action* of these bodies is as perfect as the analogy of *condition* appears to be, and, whether, "when the process of respiration is modified by contact with a matter in the progress to decay, when this matter communicates the decomposition, of which it is the subject, to the blood—disease is produced."

We shall first state the analogy of condition of the *tangible poison*, evolved from decomposing organic substances, in the words of Dr. Southwood Smith—not only because it is clearly stated by him (so far as relates to its tangible existence), but, also, because this passage has furnished the text for some of the objections which we shall have to consider*:—"It is known to every one that the putrefaction of vegetable and animal matter produces a poison, which is capable of exerting an injurious action on the human body. But the extent to which this poison is generated, the conditions favourable to its production, and the range of its noxious agency, are not sufficiently understood and appreciated. It is a matter of experience, that during the decomposition of dead organic substances—whether vegetable or animal—aided by heat and moisture, and other peculiarities of climate, a poison is generated, which, when in a state of high concentration, is capable of producing instantaneous death by a single inspiration of air in which it is diffused. Experience also shews that this poison even when it is largely diluted by an admixture with atmospheric air, and when, consequently, it is unable to prove thus suddenly fatal, is still the fruitful source of sickness and mortality—partly in proportion to its intensity, and partly in proportion to the length of time, and the constancy with which the body remains exposed to it, &c.

"But this poison was too subtle to be reduced to a tangible form. Even its existence was ascertainable only by its mortal influence on the human body; and although the induction commonly made as to its origin, namely, that it is the product of putrefying vegetable and animal matter, appeared inevitable, seeing that its virulence is always in proportion to the quantity of vegetable and animal matters present, and to the perfect combination of the circumstances favourable to their decomposition, still the opinion could only be regarded as an inference.

* Poor-law Commissioners' Fourth Report, page 130.

But modern science has recently succeeded in making a most important step in the elucidation of this subject. It has now been demonstrated by direct experiment, that in certain situations, in which the air is loaded with poisonous exhalations, the poisonous matter consists of vegetable and animal substances in a high state of putrescency. If a quantity of air in which such exhalations are present be collected, the vapour may be condensed by cold and other agents, a residuum is obtained, which, on examination, is found to be composed of vegetable or animal matter in a high state of putrefaction. This matter constitutes a deadly poison. A minute quantity of this poison applied to an animal, previously in sound health, destroys life with the most intense symptoms of malignant fever. If, for example, 10 or 12 drops of a fluid, containing this highly putrid matter, be injected into the jugular vein of a dog, the animal is seized with acute fever, the action of the heart is inordinately excited, the respiration becomes accelerated, the heat increased, the prostration of strength extreme, the muscular power so exhausted that the animal lies on the ground wholly unable to stir or to make the slightest effort, and after a short time it is actually seized with the black vomit, identical in the nature of the matter evacuated with that which is thrown up by a person labouring under yellow fever. By varying the intensity and the dose of the poison thus obtained it is possible to produce fever of almost any type, endowed with almost any degree of mortal power."

In this last sentence we recognise the echo of Majendie's questionable assertions; the preceding statements are confirmed by the account of experiments upon "le mauvais air," given by Devergie:*—"The gas, which is disengaged from putrefying animal matters, extracts with it a particular odour, infectious 'infecte,' characterized by the general term putrid odour. We attribute this odour to miasma, that is to say to a cause void of meaning, because we are ignorant of the nature of the object which it represents.

Guntz has endeavoured to enlighten the phenomenon by the following experiment: he placed a bell glass over a portion of a putrefying dead body, in such a manner as to permit the air to penetrate, he submitted the apparatus to a temperature of 26° Cent. (equivalent to about 78° Far.) and, after a period sufficiently prolonged, he suddenly cooled the bell glass; immediately the product of the vapour assembled itself into drops, which evolved a strong odour of miasma, he treated these drops with chlorine, when the odour disappeared. He was thus led to suppose that the gas in escaping from the putrefying animal matter carried with it the vapour of water combined with a certain quantity of animal matter, very minutely divided, and this constitutes what has been named miasma.

This is not the only experiment calculated to lead to this opinion—others have been made with respect to vegetable matters. Moscati entertained the first idea of condensing the water dissolved in the atmosphere, for the purpose of detecting the principle which occasioned "le mauvais air." He suspended at some distance from the soil mattrasses full of ice; the water which became deposited upon their surfaces condensed itself readily, when limpid it presented many small flakes which possessed all the essential properties of *animalized matter*. After a few days they putrefied completely. In the course of the year 1812, M. Rigaud undertook, in the marshes of Languedoc, a series of essays directed to the same end. He condensed dew on glasses, and the water which he obtained by this means presented all the phenomena obtained by Moscati.

In 1819 M. Boussingault observed that *sulphuric acid* placed in the proximity of a well, in which he had caused animal matter to putrefy, *blackened* very rapidly. He repeated this experiment in many infectious places, and found con-

---

* Medicine Legale. Tom. 1, p. 100. I am indebted to my friend Dr. Aldridge for referring me to this account of experiments on the subject.

stantly that the coloration of the acid was more prompt according as the air was more infectious," &c.

The inference naturally deduced from such experiments as the foregoing, taken in conjunction with the fact of the occurrence of fever in situations where these putrid exhalations have been found to exist—namely, that they contain a *fever poison*—has been met by numerous objections. The principal seem to be the following:—1. That a mephitic poison is confounded with the fever poison. 2. It is denied that these sources ever generate fever, because the number of cases does not bear a sufficient proportion to the number of instances of exposure, and because they generate several diseases differing in their nature from fever, and it would amount to a confounding of fever with these,* if we attributed its origin to the same poison. 3. It has been objected to the evidence of the frequent occurrence of fever from this source—that it is furnished from the experience of persons who deny the infectiousness of fever, and is, therefore, suspicious; † that in the recorded cases malaria and contagion have been confounded; that it amounts only to a proof of the frequent coincidence of fever and the effluvia from filth, and does not prove that the former stands to the latter in the relation of an effect to a cause; that, granting continued fever is ever thus produced, it is not contagious or typhus fever, &c.

I. It has been said‡—"If the statements of Dr. Smith were put into the simple form of the only proposition which they really contain, they would amount merely to this—that exhalations from certain putrescent matters have the power of producing both asphyxia and continued or typhus fever; the former of which is a result familiar to all, and the latter, a mere assertion, deriving a little hue of probability from its juxta position to a known truth. There is a wide difference between the asphyxia, which is caused by mephitic gases, and typhus fever,—a difference which can never be explained, as Dr. Smith attempts to do, by a reference to the diversity in the doses of the poison. We presume that if a few doses of the poison, in its less potent shape, were sufficient to create typhus fever, *a fortiori*, such a quantity of it in a more concentrated form, as would be capable of producing a state of asphyxia not ultimately fatal, would commonly at least leave the sufferer for days or weeks in the toils of a highly dangerous fever, yet the reverse is the case, as the histories of mephitism amply demonstrate."

So they doubtless do, and they, moreover, shew that in some cases of recovery from mephitism, a disease, apparently the effect of a morbid poison, followed, though *not* fever.

But this writer has, like Dr. Smith, confounded the action of two poisons of different kinds—an inorganic poison, sulphuretted hydrogen, and a morbid poison, whose action depends not upon its chemical qualities, but upon the existing condition of its particles, they being at the time of their evolution in a state of decomposition or transposition.

The advocate for the malarial origin of fever does not regard the fever poison as the product of *extreme putrefaction*, capable of causing mephitism or fever, according to the dose in which it is applied. But he holds that during the *progress* to decay of organic substances, matter in a state of decomposition is

---

* "Dr. Smith illustrates and supports his doctrine of the malarial origin of fever by referring to facts which relate merely to periodic fevers; and he maintains the identity of the 'fever poison' of this country with the poison of plague; wherefore, on the principle, that things that are equal to the same thing are equal to one another—plague and ague are generated by the same poison!"—*Forbes's Review*, No. 21, p. 13.

† Vide Dr. Christison's article—Fever. Library of Medicine.

‡ Forbes's Review, ut supra.

evolved which is capable of communicating its state to the organism with which it may be brought into contact, while, on the completion of the process of decomposition, the morbid poison ceases to be evolved and the mephitic poison is generated.

A perfect analogy to this is found in the effects of decayed sausages, which, according to Christison, "are poisonous only at a particular stage of decay, and cease to be so when putrefaction has advanced so far that sulphuretted hydrogen (the mephitic gas) is evolved." True, in mixed sources, and those which are receiving daily additions of new matters, the morbid poison may co-exist with the mephitic poison, and the latter may occasionally, by its sedative effects upon the nervous system, assist the operation of the former; but they are essentially different in their nature and action.

The second objection—"an alleged want of proof that the fever poison is ever generated in such sources"—rests, 1st, upon the relatively small number of cases of fever so produced, compared with the activity of contagion; and, 2ndly, upon the fact that several diseases of different kinds, from tic-doloureux up to plague, are attributed to miasmic effluvia. Can we (it is asked) believe that they are all owing to the same poison?

The first of these grounds is urged against decomposing animal matters; the second chiefly against mixed sources, as sewers, banks of rivers, &c.

It is true that very few observations exist which can be said to prove the occurrence of fever from exposure to animal putrefaction—still, some such cases have occurred. The following is referred to by Dr. Christison as an unexceptionable one:—

*"An American merchant-ship was lying at anchor in Wampoa road, sixteen miles from Canton. One of her crew died of dysentery. He was taken on shore to be buried; no disease of any kind had occurred in the ship from her departure from America till her arrival in the river Tigris. Four men accompanied the corpse and two of them began to dig a grave. Unfortunately they lit upon a spot where a human body had been buried about two or three months previously, as was afterwards ascertained,—the instant the spade went through the lid of the coffin a most dreadful effluvium issued forth and the two men fell down nearly lifeless: it was with the greatest difficulty their companions could approach near enough to drag them from the spot and fill up the place with earth. The two men now recovered a little, and, with assistance, reached the boat and returned on board...... One of these men died on the evening of the fourth day, and the other on the morning of the fifth, after symptoms of malignant petechial fever (the petechiæ occurring on the fourth day).

"In eight days after the opening of the grave one of those who were not engaged in the work was attacked with the same symptoms as his companions, and the fourth had a slight indisposition of no very decided character."

It is to be remarked, that in the above case two circumstances were present which we shall see have not always existed in the negative instances, brought forward to prove that this is not a source of fever; namely, "*confinement* of the effluvia," and a *not very advanced* stage of putrefaction. Ferriar, who did not consider the exhalations from putrid animal matters a source of fever, says—†"It appears from some late observations made on altering the vaults of a church in France, that the *confined effluvia* of putrid bodies produce fever when brought into action. Perhaps this is the solution of the question." ‡"Fourcroy states, that the grave-diggers informed him that the putrid process disengages

* Medico-Chirurgical Review, for Jan. 1825, p. 203. Dr. Christison also refers to the Mem. de la Soc. Royale de Med. 1. 97.

† Medical Histories, vol. 1. On New Contagions.

‡ Walker's gatherings from Grave-yards, p. 124.

elastic fluid, which inflates the abdomen and at last bursts it; that this event instantly causes vertigo, faintness and nausea in such persons as, unfortunately, are within a certain distance of the spot where it happens, &c." In the exhumations, conducted on such a large scale at the cemetery of the Innocents, and quoted by Bancroft and others in proof of his position, neither of these conditions could have existed, since no interments had been allowed for six years previously.

In many of the cases also related by Mr. Walker it is mentioned that the bodies had been buried for years, or were in an advanced stage of putrefaction; under these circumstances mephitism was produced—but not fever.

The other part of this objection, namely that so many different diseases are ascribed to this source—can they all be the effect of the same poison? can only be answered by supposing a variety of morbid poisons to be formed together or consecutively in the same source. Several considerations render it probable that this is the case in some malarial sources.

1. The progressive nature of the changes which the decomposing body undergoes, and the different circumstances under which the same organic matters (undergoing decomposition) may be placed in different places, or at different times. There is nothing improbable in the supposition, that the same source may at one time give origin to the poison of ague, and at another to the poison of fever.

2. The fact that an individual exposed to these sources will frequently become affected with two diseases. These will usually follow one another at a short interval. " In the vast horde of cases," says Dr. Addison,* "which the river side is continually sending forth, synochus and typhus are of frequent occurrence, and these are frequently followed, when the patient is convalescent, by well-defined agues." Sir H. Marsh has noticed the same occurrence in the epidemic of 1826, in Dublin, and it is well known that after this epidemic the hospitals of that city were filled with cases of ague. This is perfectly analogous to what happens from exposure to two morbid poisons, the one which has the shortest latent period takes precedence and is followed by the other, as in the case related by Dr. Williams of a boy who was inoculated at the same time with the virus of measles and cow-pock. The cow-pock first ran its course, and was then followed by measles. In the same way it is very possible that the poison of ague, imbibed at an early part of the year, may lie latent until the conclusion of a continued fever received many weeks later.

3. This power of generating different diseases, is alleged *of* those sources in particular which contain a *variety* of organic matters, and which are in a state of constant change from the superaddition of new materials or from atmospheric changes—such are sewers, the banks of rivers, &c.,—and it is to these that the great body of evidence, as to the frequent production of fever, applies, and not to the regular, uniform, and spontaneous decomposition of any single portion of animal matter, however great its bulk.†

---

* On Malaria. London Medical Gazette,—vol. 3, new series.

† If the subject admitted of an explanation purely hypothetical we might draw an analogy, not destitute of plausibility, between the action of human contagion and putrefaction in this particular; and we might suppose, that as in fever generated spontaneously in the human body, there does not seem to exist any power of communication by infection, *so* in dead animal matter the product of any single mass of spontaneous putrefaction is not a fever poison, but that this is generated by the exposure of *fresh* dead organic matter to the contagion of the former. For this hypothesis to be consistent with the facts, the following should be the various consequences of exposure to putrefactive decomposition:—

SECT. II.—In order to obviate the objections urged against the evidence of the frequent occurrence of fever from malarial sources,—namely, that it has been confounded with contagion, and that, at all events, the evidence proves, no more than the frequent co-existence of filth, fever, and poverty.

We shall select only a few cases which have occurred under circumstances unfavourable to the supposition of such a cause as contagion, and the histories of which present contrasts to that of contagious fever in some of the following particulars.

I. The class of persons affected, not those usually obnoxious to contagious fever unless under circumstances of prolonged exposure.

II. Occurring without the presence of any of the aids to contagion and at an opposite season of the year.

III. In localities in which contagious fever does not prevail.

IV. Spreading in spite of the preventive measures, which are found to check the diffusion of contagious fever.

"In great towns," says Christison, "cases are met with during the intervals between epidemics, and in a station of life where epidemic fever in epidemic seasons of the worst kind is seldom witnessed. A fever of this description, tedious in its course, characterized by much nervous and muscular depression, without any particular local disturbance, and, especially, without the marked disorder of the functions of the brain which distinguishes most cases of epidemic typhus and synochus, was so prevalent among the better ranks in certain streets of Edinburgh some years ago, at a time when fever was not prevalent among the working classes, that a general impression arose among professional people of the existence of some unusual local miasma. A great variety of parallel facts might be referred to—all leading to the general conclusion, that a disease if not identical with, at all events closely resembling, synochus and typhus as described above, may arise without the possibility of tracing it to communication with the sick. A statement of this kind acquires great weight in the instance of such a visitation of disease as that just alluded to, which prevailed among people in easy circumstances in a great town."

Very similar is the testimony of Dr. Cheyne :—"For several years the fever appeared in families only in solitary instances, or if more than one were affected they were seized nearly at the same time, but it did not extend so as to lead us to think that it propagated itself. We were unable to assign the cause of the disease further than that we observed in several houses, in which our patients lay, that fætor which is discoverable when a sewer is choked, and, in some instances, upon enquiry it was found that the sewer leading from the house had been improperly constructed and neglected."

A similar instance of fever, apparently caused by defective seweiage, came under my observation recently in the house of a gentleman of fortune in this county. For a long time an unpleasant odour had been remarked in seveial parts of the mansion, more especially near this gentleman's study and in the

---

1. From exposure to a single mass of animal matter undergoing spontaneous putrefaction—no fever.

2. From exposure to the emanations from substances added to the former disease, varying in intensity in proportion as circumstances were more or less favourable to rapid communication of the "contagion of decay" from decomposing to recent organic matter.

3. From successive exposure of a number of individuals to successive additions of organic matter, (under above circumstances), a number of cases of disease.

men servants' sleeping apartment. The poisonous effects of the malaria were first produced in the form of obstinate dysentery in one of the female servants. Then the owner of the mansion was attacked with what he at first supposed was mere biliary derangement, but which rapidly assumed all the characters of severe gastric fever, becoming attended toward the close with purple petechiæ and terminating fatally on the 11th day.

About the same time two men servants were seized with symptoms of fever. In one it was cut short and in the other it ran its course, ending favourably about the 11th day. Two other persons who came to the house on business (from the neighbourhood) and who remained in it for a few hours, were seized with the same fever, which ran through its course at their own houses but without extending to other individuals.

After this lamented occurrence the cause of the effluvium was searched for and found to be a leakage of the soil pipe of one of the water-closets, which had allowed the filth to percolate through the wall and exhale into the atmosphere of the house. This exhalation was also much favoured by the warm temperature kept up in the house by heated flues.

About the months of October and November, 1839, I was repeatedly consulted by the inmates of a large establishment in the neighbourhood of Navan, on account of different forms of gastro-enteric affection, especially diarrhœa and dysentery. So many instances occurred at intervals, (in some cases of weeks), and the general resemblance was so great, that I thought they must arise from some local cause, and I expressed a strong suspicion that some source of malaria existed in the house or immediate vicinity. The house itself was large, airy, and commodious, so that our inspection was directed rather to the immediate neighbourhood, and it was thought that the cause had been discovered in an old sewer which had been laid open in the course of some building operations. The closing of this was not, however, attended with the effect of stopping the endemic affection, though it gradually ceased after about a dozen people had been attacked. The following spring was remarkably dry, scarcely any rain having fallen for about six weeks ; toward the close of this period an effluvium of a very disagreeable nature became perceptible in some parts of the house, and at the same time —within a day of each other—two of the inmates were attacked with exquisitely marked typhus, attended with profuse measly eruption, and in one of the patients with violent delirium. Every circumstance rendered the existence of contagion in either case highly improbable, I might almost say impossible, and on my again expressing my strong conviction that some form of malaria was the cause of the fever, I was informed of the effluvium perceived in some of the passages, and also of the fact that in the original construction of the water-closets they had been made to depend for their supply of water upon a cistern of rain water, which, of course, had been for weeks empty during the present spring and preceding autumn. These cases did not spread, and all traces of indisposition were removed by making the required alterations for ensuring a constant supply of water.

In the month of October, 1839, I attended a respectable man who resided in a large and airy mansion, as "care taker," during the absence of the family upon the Continent. His illness had come on slowly and insidiously, but, when I saw him, had all the characters of bad remittent fever, attended with much abdominal congestion. This it was attempted to relieve by leeches, &c., but it increased and led afterwards to large evacuations of blood from the bowels. He recovered slowly and with difficulty. At the time I saw him he was lying in the basement story of the house.

A few weeks after the return home of the family, the *butler* was attacked with symptoms of gastro-enterite and slight jaundice. He recovered partially in a few days, but in the course of a week after was suddenly seized with complete loss of muscular power, paleness and coldness of the surface, sickness of stomach, &c., followed by vomiting of a dark olive fluid, and in two days by large evacuations of tarry blood from the bowels, hiccup, subsultus, &c., while the skin was covered with vibices and black petechiæ—some of them of the size of large shot. The fever which followed had no perceptible remissions and perfectly accorded with the descriptions of putrid malignant fever by Huxham and others. The striking resemblance of some of the symptoms of this case to that which had occurred in the same place more than a year before, led me to attribute both to a common cause and to enquire for the source of the malaria. The following were the facts ascertained :—In a room in the basement story, occupied by the last patient and in which he had latterly sometimes slept, was a sink emptying into a pipe, which communicated at the distance of about ten feet with the main sewer of the house—into which the contents of two water-closets passed. This sewer was very large at its termination and when the wind blew from that direction towards the house, there being no smell trap under the sink, the effluvium of the sewer was carried up into this room and became so insupportable that the patient used to stuff the aperture with a piece of rag when retiring to bed. Upon enquiry I was informed that the first patient had frequently before his illness remarked the same fœtid effluvium. It is worthy of remark that the sewer had *not* been cleaned in the interval between the two cases.

The following case, very similar to the last in its nature and origin, I give upon the *suspicious* authority of an anticontagionist* :—" I attended," says Dr. Armstrong,† " a very respectable tradesman, labouring under a remarkable bad attack of typhus fever. It was such a case as would have been called plague in the time of Sydenham. He had knotted glands and carbuncles, and black petechiæ. He was one of four or five individuals who had transacted some business in a nobleman's kitchen ; a filthy fluid had overflowed that kitchen ; he was sickened at the time, and in common with *all* the others had an attack of typhus fever."

If we looked about for a large town less liable to contagious fever than others we might probably find it in Birmingham,—yet here endemial causes of the kind, which Dr. Davidson has pronounced inadequate to this effect, have produced fever. A good instance for illustration is found in Dr. Ward's account of an endemic fever, which prevailed in certain localities in Birmingham in the *summer* of 1837.‡

"The river Rea, that separates Birmingham from its suburb Badesley and serves as a cloaca maxima to both, carries its filthy stream onward, partly to turn a mill and partly to fill a mill pond. During the drought which prevailed last year the water was very low in the main stream and mill pond, and the mills not being regularly worked became quite stagnant and offensive. The back stream also became dry and shewed its mud banks, that were only occasionally wetted by a flush of the washings of the town after a shower, or by the small surplus accumulated during the cessation of the working of the mills. The exhalations from the half dried mud and putrid water were so disagreeable at night as to nauseate the more delicate inhabitants of the adjoining streets, and soon produce disease in the form of typhoid fever of an infectious (?) character." He goes on to state that about 50 cases—some fatal—occurred in the immediate vicinity of the stream, and "still lower down the stream, where the water was as black as ink, there were 13 pauper cases in one yard, and many others, both pauper and private, along the same line." That this fever was owing to the state of the stream is proved by the disease being confined to the locality, the small number affected in so large a population as Birmingham, the season of the year, and the exemption of this town from the causes which aid contagion —these are well-summed up by Dr. Ward.

* See Christison, ut supra. † Lectures by Rix.

‡ Provincial Medical Transactions, vol. 6.

"There is a difference of nearly 200 feet in the elevation of different parts of the town. The streets and the courts or yards in which the mechanics live are wide and airy in general; fuel is cheaper than in any other large town in England; the water is excellent—and till within the last year there has been but little distress."

We have already adduced the effects of seclusion of the sick in proof of the infectiousness of typhus. In the fever arising from endemial sources this measure has no such influence. I was much struck with this fact when making some investigations as to the source of a fever, which prevailed in the summer of 1839 in a hamlet attached to a flax manufactory near this town, from which a considerable number of cases had been sent to hospital in the months of April, May, and June. The object of an examination which I made of the place was to obtain satisfactory instances of contagion, but I soon found that no such evidence was to be procured. For the intervals between the illness of different members of the families were too irregular to admit of communication from one to another. Thus, in one house the first case sickened on the 2nd of April, and the second on the 5th. In another, more than three months intervened between the first and second cases. And in several families in which the first case had been early removed to hospital, the second had sickened before the patient's return. Besides, there was too much cleanliness and comfort: several of the houses had been repeatedly white-washed during the time that the fever was going through the family, and the inmates were all well off—being employed in the neighbouring factory.

Several things convinced me that this fever had a malarial origin. The hamlet was built in the form of two parallel streets, terminating in a large open space, in front of which were twelve houses looking North East. This space had no drainage and was full of shallow pools of black putrescent water, into which the inmates daily threw cabbage leaves, &c. to rot for manure. In this country the East and North East winds prevail for the first three months of summer—April to June—and in consequence the inhabitants of the twelve houses described were peculiarly obnoxious to the emanations from these pools. The weather had during the summer been unusually dry and favourable to such emanations. Accordingly I found that while only seven cases of fever had occurred in twenty-two houses, forming the longer of the two streets, 30 out of 50 (the entire number) had occurred in these twelve houses.

The proof was to my mind rendered complete by the immediate effect of the heavy rains which set in in July. The disease was stopped at once and I have not heard of a case of fever in the same place since.

Similar proof, derived from the sanatory effect of the removal of the assigned cause, was afforded me in the case of a house in this county from which three servants were sent into the Navan hospital at short intervals labouring under continued fever, one of whom was also admitted a second time with severe dysentery. A very offensive smell had been long noticed in the yard adjoining the kitchen, and after the occurrence of these cases the sewer leading from the kitchen was in consequence examined, and found to be completely obstructed by a quantity of black putrescent matter. Upon the removal of this the smell of course disappeared and no return of indisposition has since occurred, either among the servants or family. There was not the slightest evidence of contagion in any of these cases.

A reason for attributing the fever to the operation of endemial causes might be found in some instances, in the fact of the great indisposition of the disease to spread in the house in which a case occurs, even though the circumstances favourable to contagion may be present. Such an instance is given by Dr. Fergusson which we shall have again to notice.

In a paper on the statistics of fever, in Belfast, Dr. Mateer states that "one street, Carricks Hill, and its continuation, Mill-field, with the adjoining lanes and entries, are found to have furnished three fifths of the whole amount of cases, and yet they are by no means the poorest or worst ventilated parts of the town." He attributes the prevalence of fever in this locality to the great want of water—"the consequences of which are want of cleanliness and bad sewerage, so that decayed animal and vegetable matter of all kinds, not being carried off by a current of water in the usual way, accumulate and generate miasmata."* This observation is of the more value that Dr. Mateer adduces it in support of the action of infection. Upon which an acute reviewer remarks :—"Surely this offers the very strongest argument against Dr. Mateer's own view of the extensive operation of contagion ; why should this be all powerful in one particular locality ? why should it not do its worst where poverty and bad ventilation flourish ? why, but that on the large scale other causes of fever are far more potent than contagion." †

Lastly, the following case by Dr. Currie illustrates this form of fever in several particulars :—

"The 30th Regiment, as is usual with troops in Liverpool, was billetted in the town but paraded and mounted guard in the fort, situated north of the town and on the banks of the river. The general guard room had been used, previous to the arrival of the 30th, as a place of confinement for deserters; it was extremely close and dirty, and under it was a cellar, which in the winter had been full of water. *This water was now half evaporated and from the surface issued offensive exhalations.*

In a dark, narrow, and unventilated cell off the guard room it was usual to confine such men as were sent to the guard for misbehaviour, and about the beginning of June, 1792, several men had been shut up in this place on account of drunkenness, and suffered to remain there twenty-four hours under the debility that succeeds intoxication. The typhus or jail fever made its appearance in two of these men about the first of the month and spread with great rapidity. Ten of the soldiers labouring under this epidemic were received into the Liverpool Infirmary...... The symptoms of the fever were very uniform, in every case there was more or less cough with mucous expectoration ; in all those who had sustained the disease eight days and upwards there were petechiæ on the skin, in several there were occasional bleedings from the nose and streaks of blood in the expectoration. The debility was considerable from the first...... Great pain in the head with stupor pervaded the whole, and in several instances there occurred a considerable degree of low delirium...... Our next care was to stop the progress of the infection ; with this view the guard house was at first attempted to be purified by washing and ventilation ; the greatest part of its furniture having been burnt or thrown into the sea. All our precautions and exertions of this kind were, however, found to be ineffectual. The weather was at this time wet, and extremely cold for the season ; the men on guard could not be prevailed on to remain in the open air and from passing the night in the infected guard room, several of the privates of the successive reliefs on the 10th, 11th, and 12th of the month, caught the infection...... No means having been found effectual for the purification of the guard room it was shut up, and a temporary shed erected it its stead. Still the contagion proceeded on the morning of the 13th, three more having been added to the list of the infected. On that day, therefore, the whole regiment was drawn up at my request, and the men examined in their ranks : seventeen were found with symptoms of fever upon them. It was not difficult to distinguish them as they stood by their fellows. Their countenances were languid, their whole appearance dejected, and the admata of their eyes had a dull red suffusion. These men were carefully sepa-

* Dublin Medical Journal, September, 1836.
† Medico-Chirurgical Review, Oct. 1836.

rated from the rest of the corps and immediately subjected to the cold affusion. . . . .These means were successful in arresting the epidemic—after the 13th of June no person was attacked by it."

It may seem presumptuous to offer an opinion differing from that expressed by the distinguished writer under whose observation these cases occurred, but we think there is every reason to question the existence of infection, and to regard them as of purely endemial origin. Let us consider them with reference to the circumstances unfavourable to the existence of contagion before enumerated.

1. *The class of persons affected.* British soldiers in time of peace are not obnoxious to contagious fever. The fact is stated by Dr. Cheyne, that while fever of this kind prevailed in the street contiguous to the principal barrack in Dublin, in 1817, and among a class of persons with whom the soldiers commonly associate, they escaped, because "little under the influence of the predisposing causes of fever; for the pay of the soldier is ample, he is well clothed, well fed, well lodged, and well looked after, and all his wants in health as well as in sickness are provided for." *

2. *The season of its occurrence* is another strong reason for considering this fever of an endemial kind. A contagious epidemic may live out the summer, but unless it is imported we should doubt its being generated at that season.

3. *The locality* was also unfavourable. Isolated as it was, an imported contagion was unlikely.

4. *The inefficacy of all the preventive measures,* short of removal from the locality, with the immediate cessation of the disease which followed this step, are strongly opposed to the idea of infection. In fact if it be admitted that the stage of maturation or crisis is the period of infection, an examination of the dates of these cases will shew that in no one was the disease so far advanced as to have enabled the patient to communicate it to his comrades, supposing them (which is not at all probable) to have had access to the hospital. On the other hand the positive evidence in favour of malaria is clear and decisive. Several individuals were exposed to this source during the debility which succeeds intoxication, and slept in its immediate neighbourhood. They were attacked, and others in succession as they became exposed to the same source. The malarious spot is abandoned on the 12th, and no case is observed after the 13th. Hereafter we shall attempt to shew that the symptoms of these cases were such as characterise not typhus but typhoid fever—especially the late appearance of petechiæ, the exudations of blood from the air-passages, and the form of disturbance of the sensorial functions.

The above are a few of the instances which might be brought forward to prove the occurrence of fever in situations and circumstances unfavourable to contagion, and not liable to the objection that *filth* has existed merely in fortuitous connexion with *fever and poverty.* It would not be difficult to draw from the published histories of infectious fever (so called) such a number of similar facts, as would render doubtful the justice of Christison's objection, that "as for the few instances remaining, where true primary fever appears to originate in one of the above causes, all that need be said farther is that for one instance where such fever follows such cause, a thousand instances occur where no effect of the kind ensues, and that, consequently, some more essential influence is probably brought into play, than what appears merely on the surface of the investigation." But some argue that the disease produced is not fever—for, first, it does not diffuse itself as fever does by infection. This is not the place to enter upon an examination of the conflicting statements upon this question—we shall do so hereafter; but admitting that it has been asserted too hastily by Dr. S. Smith, and others, that *infectious fever* is generated by paludal sources, we

* Dublin Hospital Reports. Vol. 2.

deny that this justifies the inference sought to be deduced, that, therefore, *continued fever* is not so generated. On the contrary, it seems more consonant with reason to infer, that if fever affects a number of individuals in a certain locality without appearing to be communicated from one individual to another, and without, in any instance, being carried from that locality, this fever must arise from some local source common to all the affected persons. And if that party are in extreme, who hold that fever of a contagious specific character is daily generated by common causes external to the human body, equally so are the opposite party who deny to these sources the power to cause fever, "not typhoid," while at the same time they are ready to admit the identity of their own "specific contagion" with a disease which, the most eminent observers maintain, is never contagious! It is surely more consistent with the doctrine of the speciality of typhus to let it stand alone, and to give a place to non-contagious continued fever, than to exclude the latter by a doubtful assimilation of typhus and typhoid fevers. Hereafter we shall attempt to shew that the most recent science is in accordance with the practical observation of Grant, that "these fevers, 'typhus,' are generally contagious which the common fevers are not, unless their nature is altered, and they are rendered malignant *by bad treatment*"—while we may see reason in the present state of society in our large cities, in the widely prevailing influence of crowding, poverty, non-ventilation, &c., and the consequently frequent and facile transition of *common* into *contagious* fever, why the most opposite conclusions are formed as to their origin and diffusion, and why it happens, as Christison truly remarks, that "the greater proportion of the discrepant doctrines of the present day as to the origin of fever are founded essentially upon the same great body of facts."

Again, by some it is urged that the disease produced by paludal emanations differs from continued fever in symptoms and in type. Thus Dr. Christison alleges that "few inquirers have taken sufficient pains to distinguish primary continued fever from irritative gastric fever." This objection cannot be allowed to have much weight so long as the *primary* nature of typhoid fever is a matter of dispute. Upon the subject of the type Dr. Christison may be quoted against his party, for if, as he asserts, "The coast remittent fever of Africa and other tropical countries seems to differ little in its characters from synochus, with a rapid and early stage of typhoid depression,"* what becomes of the argument against the malarial origin of continued fevers from alleged differences in the nature of these and the intermittent and remittent fevers, also produced by malaria? Besides how can the exclusive contagionist answer the anti-contagionist who rests his doctrine on such facts as those adduced by Armstrong: "Shortly after I had published my 3rd edition on typhus fever, in which I had strenuously maintained the doctrine of human contagion, I met with a case of intermittent fever; in a few days the fever became remittent, and in a few days more put on the continued character, and the patient died with all the most malignant symptoms?"† or how will he dispose of the assertion of Dr. Elliotson, that most cases of so called typhus fever are really remittent, ‡ or explain

---

* Library of Medicine. † Lectures by Rix.

‡ Lectures by Rogers. Dr. Mateer also observes—"We have the paroxysms of which fever is made up best seen in the intermittent and remittent fevers, but still by careful observation we can detect something of the same kind, though masked and often difficult to recognize, in the continued fevers of this country. These almost always assume more or less of the remittent character."—*Dublin Journal, ut supra.*

Dr. Currie remarks, whoever has watched the progress of fever must have observed the justness of the observations made by Cullen, Vogel, De Haen, and others, that even those genera which are denominated continued are not strictly the occurrence (already noticed) described by Marsh and Addison of well-defined ague, following on the subsidence of continued typhus or synochus? Was the ague also the effect of contagion? Or will the contagionist escape from the necessity of adopting so easy a solution of the difficulty as the supposition of different morbid poisons, generated at different periods in the same locality by a simple denial of the fact, and an impeachment of the accuracy of the observers who have recorded it? "In Sydenham's time," says Dr. Hancock, "and even in that of Fothergill, the quotidian of spring became continued fever in summer, while the simple continued fever of summer often changed to a malignant type in autumn. These were simple observations at a time when systematic arrangements had not put physicians in trammels. But now lest we should be guilty of medical heresy we must not insinuate that ague can change into continued fever, and non-contagious fever into contagious typhus, either in an individual case or in the course of the year."

SECT. III.—*Varieties of the Sources and Modes of Application of the Poison.*

The organic matter constituting the source of the morbid poison may be purely animal, vegetable, or a mixture of both.

It must be admitted that fever seems to be very rarely produced by exposure to purely animal exhalations, and the facts brought forward by Bancroft, Chisholm, Duchatelet, and others, shew that in the great majority of cases this exposure has been continued for any length of time with perfect impunity, but still there have occurred well authenticated instances to the contrary, some of which have been referred to; and a fact lately published, by M. Devergie, deserves farther notice. It is the occurrence of hospital gangrene in the hospital of St. Louis, which he attributes to the emanations from Montfauçon, since the disease was confined to the wards which were exposed to those emanations, and did not appear in other parts of the building.

Now if we admit the inference which seems naturally to follow from such instances as those related by Pringle, Hennen, &c., of the occurrence of typhus in the unwounded in wards, in which hospital gangrene existed, and of typhus attacking the attendants employed in washing the bandages of the same—namely, that hospital gangrene is a modification, or as it has been expressed, "a visible personification" of the typhus-poison; we cannot avoid the admission that a fever poison may be generated by decomposing animal matter under certain conditions.

What the conditions required for this result may be, and why it so seldom happens that fever is thus produced, are questions to be resolved by deeper and more accurate investigations than appear yet to have been made.

There seems to be a more general belief in the activity of the vegetable poison, though why it might be difficult to say, unless from juxta-position with the known fact of their power to cause periodic fevers, since there is at least an equal paucity of strict evidence with regard to this as to the animal source. About 15 years since I witnessed the origin of a highly typhoid petechial fever in a healthy village in England, which appeared to arise from a vegetable source

---

such, but have pretty regular and distinct exacerbations and remissions in each diurnal period.—*Med. Reports*, p. 16.

And Dr. Fordyce, says the similarity between these three kinds has determined practitioners of the greatest eminence, through the whole history of medicine, to consider them as the same disease. Many have thought that in a continued fever the subsequent paroxysm takes place in the hot fit of the prior paroxysm, &c.—(3*rd Dissertation*, p. 59.)

a heap of putrefying turnips. In a house close to the nuisance, a boy had for two or three weeks been complaining of headache, lassitude, and debility, but had not been placed under any medical care. On the day on which I saw him he had been attacked with epistaxis which continued till his death, on the day following. His skin was covered with small ecchymotic petechiæ. After his death petechial fever appeared in the family, consisting of six persons, and in the adjoining houses, and proved fatal in several instances. It did not spread beyond the locality and subsided in a few weeks. The season of the year (summer), with the other circumstances, were unfavourable to the supposition of contagion.

But it is to sources containing *mixed* organic matters that the experience of all observers point as most efficient in producing continued fever; such are slaughterhouses, obstructed sewers, cesspools, &c. &c. It were needless to add to the details already given of cases originating in these sources.

The modes in which the poison may be applied to the organism are by direct introduction into the circulation, by being taken into the stomach, by inhalation, and by the skin.

Fever, or a disease confessedly bearing a close resemblance to it, has been produced in the lower animals by experiments too well known to need us to dwell upon them, and occasionally the typhoid symptoms which appear in other diseases would seem to be owing to the absorption of putrid matters into the circulation. A case of the kind once occurred under my own observation: a boy aged ten years was received into hospital, labouring apparently under typhoid fever, he sunk after a few days, and on dissection the only lesion discoverable was a carious state of the petrous bone, with a minute opening communicating with the lateral sinus, through which the matter of the carious abscess had passed into the circulation.

It is but rarely that an instance occurs of fever produced by putrid matters taken into the stomach, and the immunity enjoyed by savages, who live much upon putrid flesh, &c., has been referred to by Bancroft and others, not only against the fact, but also against the supposition of putrid animal matters containing a fever poison. It is not difficult to understand that this should be the case, since digestion, an antiseptic process, precedes assimilation, and changes remarkably the matters submitted to its operation; or, as accurately expressed by Liebig, putrid poisons having an *alkaline* reaction are rendered inert by the acids contained in the stomach, while these exert no such power over poisonous sausages which have an *acid* reaction. But this rule is not without some exceptions. In Dr. Christison's work on poisons is a report of a case which occurred in Stockport in the year 1830, of a family of five persons who were poisoned with broth made of putrid beef; in three instances the disease produced was severe, and in one fatal. It is worthy of remark (and is in accordance with the mode of action of a *morbid poison)* that in the worst cases the illness did not commence till the second, and (in the fatal case) the third day after the meal. A case is somewhere narrated of a regiment in which putrid fever prevailed, and in which the disease was checked upon a discovery being made that the water used for drinking was drawn from a well in which some bodies were lying in a state of putrefaction.

Dr. Copland* remarks upon the effects of drinking the water of the Seine at Paris, and Dr. Hancock observes, that it has been frequently remarked that this water produces diarrhœa in every one except the Parisian accustomed to the use of it.† Dr. Tweedie refers to the history of a fever ascribed to the combined effect of drinking putrid water and the emanations from the same—and others ascribe

---

* Dictionary of Practical Medicine, art. Endemic Influences.

† Cyclopædia of Practical Medicine, art. Endemic Diseases.

the putrid fevers of Paris to the fact that "there are numerous wells in that city from which many of the inhabitants derive their whole supply of water, not a few of which are situated in the very neighbourhood where the 'fosses' are the worst constructed and the least attended to; the urine, therefore, permeating the soil must necessarily contaminate the springs from which these wells are fed."*

The evidence given before the committee on the health of towns, by Mr. J. B. Wood, bears upon this question. After stating (qs. 2150-4) that 31,000 persons live in the cellars of Liverpool—forming two-thirds of the working population—he states that, "in the districts in which these cellars are situated, there is a great deal of broken ground in which there are pits; the water accumulates in these pits, and of course at the fall of the year there is a good deal of water in them, in which there have been thrown dead dogs and cats and a great many offensive articles. This water *is nevertheless used for culinary purposes.* I could not believe this at first, I thought it was only used for washing, but I found it was used by the poorer inhabitants for culinary purposes."

The change produced in the fluids and solids of an animal by over driving seems to be capable of becoming a cause of disease in the human body. In the remarkable case given by Andral from Du Hamel it does not appear that fever, strictly speaking, was produced. The effect rather resembled hospital gangrene; but an instance is recorded of typhous fever following from eating the flesh of animals under similar circumstances. It is thus quoted by Dr. Gross: † "A few years ago a number of fattened cattle were driven into one of the New England cities, and having been pressed too hard in a sultry day were so overheated that some of them became quite exhausted. In this condition they were slaughtered, and the consequence was, as is stated by the reporter of the case, Dr. Fountain, that nearly all who partook of their flesh were seized with typhous fever."

These and similar observations would seem to shew that the morbid poison, the product of putrefactive decomposition, may be received into the system through the stomach, more especially if presented in the fluid form; but there is every reason to conclude that fever is but seldom produced in this way, and that the general mode of introduction is through the respiration of the gaseous exhalations, which, as we have seen, are found upon their being collected and condensed to contain animalized matter in the state of progress to decay, whose power of producing disease, in those exposed to their influence, has been questioned and is by many denied, but appears to be proved by evidence of a very satisfactory nature, and which applies with most force to cases occurring under circumstances unfavourable to the action of contagion.

### SECT. IV.—*On the Mode of Action of the Poison.*

This does not appear to be attributable to its chemical qualities, but to its *condition.* It is the power of communicating an action, since there can be no doubt that its effects continue to be produced equally after the removal as during the presence of the cause, when once this cause has impressed its mode of action upon the organism. What the mode of this impression is, and what part of the system is the subject of it in the first instance, we have now to enquire, as also into the order of the phenomena subsequently produced and constituting the formed disease.

The generally received opinion seems to be that the nervous system is the

* Medico-Chirurg. Review, vol. 6.
† Pathological Anatomy, vol. 1, p. 223.

subject of the first morbific impression, and its derangements the first in the morbid series constituting fever.

This doctrine is thus maintained by Dr. Southwood Smith*—"The immediate exciting cause of fever is a poison which operates primarily and specifically upon the brain and spinal chord. The diseased state into which these organs are brought by the operation of the poison, deprives them of the power of communicating to the system that supply of stimulus, (nervous and sensorial influence), which is requisite to maintain the functions of the economy in the state of health. The organs, the seats of the functions, deprived of their supply of nervous influence, become deranged, the derangement in each taking place in a fixed order and in a determinate manner.

Subsequently to the nervous and the sensorial, the organs the next to suffer are those of the circulation, then those of respiration, and, ultimately, those which belong to secretion and excretion. The condition of the nervous system, which produces this derangement in this circle of organs, occasions further, in that portion of the circulating system which consists of the capillary blood-vessels, that peculiar state which constitutes inflammation: hence inflammation is almost always established in one or more of the organs comprehended in the febrile circle and sometimes in all of them." In another passage the same writer says—"The more closely and extensively the subject is investigated the more clear and satisfactory the evidence becomes, that the great primary cause of fever is a poison, the operation of which, like that of some other poisons, the nature of which is better understood and the action of which has been more completely examined, is ascertained to be upon the nervous system. How these poisons act upon the nervous system we do not know, nor can we possibly know as long as we remain so profoundly ignorant of the nature of the action of the nervous system in the state of health."

It will be seen that Dr. Smith's argument, like that of others already examined, is rested upon a fallacious analogy to certain other poisons, and like these is open to the fatal objection, that such poisons really produce their effect upon the nervous system subsequently to their diffusion through the blood. It moreover lacks the support derived in the former cases from the occurrence of a nervous shock, since such rarely, if ever, attends exposure to the paludal sources of fever. On the other hand, some of the arguments for a modified humoral theory before adduced, apply with greater force to this than the animal miasm, and additional ones are not wanting to strengthen the proofs that all derangements of function in fever are subsequent to the introduction of the poison into the blood, and the consequent vitiation of that fluid. For, its latency is even more remarkable than that of the animal infection of typhus, while a close observation of the state of the patient, during this period, will shew a derangement of the secreting and excreting functions, which seem to be labouring to rid the system of the poison, or of those products which it has a tendency to generate in the blood; again, we can not unfrequently trace the abrupt termination of this period, and the supervention of formed disease to a suspension of the depuratory action, by cold, intemperance, or any other cause which disturbs the order of the excreting functions and arrests the elimination of the morbid product. Thus, in an exquisitely marked case of paludal fever, which was lately under my care and which terminated fatally after large evacuations of blood from the bowels, it was remarked spontaneously by the patient's friends that, for six weeks before fever commenced, not only had he suffered from capricious appetite and irregular bowels, but that a remarkable thick copious deposit had been constantly present in his urine. During this period he had been living and sleeping in immediate proximity to a collection of filth,

---

* Treatise on Fever.

which at times filled the house (in other respects a cleanly and comfortable one) with an insufferable putrid effluvium.

This instance also illustrates the cumulative property of the poison, which is much more remarkable than that of the animal infection. Unlike the latter, which is often most severe upon its first introduction into a family, the poison of malaria seems commonly to affect each successive patient more severely than the preceding.

To what can this be attributed but to the accumulation in the blood consequent upon longer continued imbibition of the poison? This is similar to the explanation offered by Cruveilhier, of the general incurability of phlebitis from the absorption of pus. Experiments have shewn that, from a single injection of putrid pus into the vein, an animal may, after copious evacuations, recover; he therefore concludes that similar success might attend the evacuant treatment of phlebitis, did not the renewal of the sources of infection follow the incessant renewal of the pus. But we can not only detect the presence and agency of the poison in the circulation through the deranged excretions, but also occasionally in the physical changes of the blood itself previous to the occurrence of formed disease. The following observations of Dr. Potter* on this subject, are highly interesting and important, and from the care and accuracy with which they appear to have been made, are entitled to great weight in the discussion of the mode of access of fevers arising from malaria. He says, " it was remarkable in all cases in which it was deemed expedient to bleed, the blood wore the same general appearances. After a separation had taken place, the serum assumed a yellow shade: often a deep orange, and a portion of the red globules was invariably precipitated.

It occurred to me that if the remote cause resided in a common atmosphere, the blood of all who had inhaled it a certain time would exhibit similar phenomena. It accorded with the pathology I had conceived, to conclude, that all who lived in an atmosphere so impregnated were constantly predisposed, and that an additional or exciting cause only would be required to develope the symptoms and form. To ascertain the appearances of the blood in subjects apparently in good health, I drew it from five persons who had lived during the whole season in the most infected parts of the city, and who were, to every external appearance and inward feeling, in perfect health. The appearance of the blood could not be distinguished from that of those who laboured under the most inveterate grades of the disease. As this experiment might have been considered inconclusive unless the blood could be compared with that of those who lived in a purer atmosphere, remote from the evolution of miasmata, I selected an equal number of persons who lived on the hills of Baltimore County, and drew from them ten ounces of blood. The contrast in the appearances was so manifest that no cause for hesitation remained. There was neither a preternaturally yellow serum, nor a red precipitate; the appearances were such as we find in the blood of healthy subjects. A young gentleman, having returned from the western part of Pennsylvania on the 10th of September, I drew a few ounces of blood from a vein on that day; it discovered no deviation from that of other healthy persons. He remained in my family till the 26th of the month, and on that day I repeated the bloodletting. The serum had assumed a deep yellow hue, and a copious precipitate of red globules had fallen to the bottom of the

* Quoted by Dr. Tweedie, Art. Fever, Cyclopædia of Practical Medicine.

Dr. Tweedie's own expressed opinion is "whether the opinion of the older writers, that in fevers originating from contagion, the contagious principle alters the properties of the blood be correct or not, we certainly think the strong analogy in the cases alluded to tends to confirm the supposition of typhoid fevers originating in diseased blood."

receiving vessel. Of the six persons whose blood assumed those indications of the remote cause, four were seized with fever during the epidemic; the other two escaped any formal attack, but complained occasionally of headache, nausea, and other indications of disease."

Similar experiments were instituted by Dr. Stevens, and with a like result, the blood being black in colour, and evidently deranged in its properties previous to the commencement of the fever.

It might almost be considered unnecessary to strengthen the inference from such facts as these, by observing on the effect upon the blood of the gases, evolved from sources which we consider the evidence already adduced proves to contain a fever poison, when these are presented in a sufficient degree of concentration to produce rapidly fatal effects.

Describing the consequences of exposure to the emanations from Parisian privies, Dr. Christison says:—"The appearances in the bodies of persons killed by these emanations are *fluidity and blackness of the blood*, a dark tint of all the internal vascular organs, annihilation of the contractility of the muscles, more or less redness of the bronchial tubes and *secretion of brown mucus there as well as in the nostrils*, gorging of the lungs, an odour throughout the whole viscera, like that of decayed fish, and a tendency to early putrefaction."

With these and similar facts before us, we cannot agree in the sweeping decision of Dr. Smith, that "changes in the fluids can only be second in the series of morbid events: they can never hold the first place in that series: they can never be antecedents or first causes, but merely sequents or effects."* We rather think that the evidence existing on the subject, if fairly examined, points to the blood as the seat of the primary operations of the morbific agent, and the subject of the changes which it is a part *of* its condition necessarily to produce. And regarding all derangements of the functions occurring in fever as the consequences of the molecular changes in that fluid, we proceed to examine into the order in which these consecutive phenomena occur—the mode of their production, and their mutual dependence one upon another.

Supposing Dr. Smith's to be a fair exposition of the views generally entertained in this country we find, upon reverting to his chapter on the theory of fever, that the doctrine maintained is, that subsequently to the supposed primary nervous impression, "the organs the next to suffer are those of the circulation, then those of respiration; and ultimately those which belong to secretion and excretion. The condition of the nervous system which produces this derangement in this circle of organs, occasions further in that portion of the circulating system, which consists of the capillary bloodvessels, that peculiar state which constitutes inflammation: hence inflammation is almost always established in one or more of the organs comprehended in the febrile circle and sometimes in all of them." If this passage admits of a precise construction it must be that in consequence of a certain impression on the nervous system a state of general inflammation exists (or at least a state approaching to inflammation) in the capillaries of all the organs, and which is equally likely to become actual inflammation in any of them during fever. Without denying this frequency of visceral inflammation in fever, or the great necessity of recognising and combatting it, it may be reasonably doubted if so variable and non-essential an occurrence—with one exception—or one so dependent for its existence and its seat upon accidental causes—as season, atmosphere, epidemic influences, states of constitution, &c.—as these local inflammations are, can be properly admitted into the discussion of a theory of fever. Either this combination of inflammation in some organ, with a peculiar state of the nervous system, is necessary to constitute fever, or it is not. That it is, seems unlikely, since morbid anatomy fails to detect it in a large pro-

* On Fever, p. 330.

portion of instances. If it be not then according to this theory, nothing will remain but a certain peculiar affection of the nervous system to account for the phenomena of fever. But in the kind of fever under consideration there is a local affection generally regarded as inflammatory, and which is so constantly present and found to exercise so great an influence on the disease as to have been considered by some eminent pathologists to be the essential cause of typhoid fever. It is that affection of the mucous glands of the small intestines described under the name of Dothin-enteritis. This affection claims a special consideration, since no one can impartially examine the evidence put forward in support of their views of the pathology of fever by Louis and Chomel, in France, or the cases incidentally published in the writings of Bright, Tweedie, Smith, Graves and Stokes, Hodgkin, &c. in this country, without being convinced that in some forms of fever—which farther examination will shew to be paludal fever—this dothin-enterite is a constant and a most important complication, if indeed it be not the pathological cause of the disease.

But if we seek an explanation of its occurrence in Dr. Smith's theory, we are first at a loss to know why an impression on the brain and spinal chord should lead to consequent inflammation in a part the most remote of any from their influence, and whose functions are under the control of a different portion of the nervous system. Then, in very many cases, we find that the symptoms of intestinal irritation preceded this nervous impression—that in others, in which death took place at an early period of fever, it was found far advanced—as by Louis so early as the 8th day—and could not therefore be regarded as a secondary phenomenon. And that in other cases, in which the poison was presented in so concentrated a form that death took place before fever could be established, the glands of Peyer exhibited the same appearance as in that disease. The following interesting case of this kind is given by Dr. Christison.

In *August* last, twenty-two boys, living at a boarding-school at Clapham, were seized in the course of three or four hours with alarming symptoms of violent irritation in the stomach and bowels, subsultus of the muscles of the arms, and excessive prostration of strength. Another had been similarly attacked three days before. This child died in twenty-five, and one of the others in twenty-three hours. On examination after death, the Peyerian glands of the intestines were found in the former case enlarged, and, as it were, tuberculated: in the other, there were also ulcers of the mucous coat of the small intestines, and softening of that coat in the colon. A suspicion of poisoning having naturally arisen, the various utensils and articles of food used by the family were examined, but without success. And the only circumstance which appeared to explain the accident was, that two days before the first child took ill, a foul cesspool had been opened, and the materials diffused over a garden adjoining the childrens' play-ground. This was considered a sufficient cause of the disease, by Dr. Spurgin and Messrs. Angus and Saunders of Clapham, as well as by Drs. Latham and Chambers and Mr. Pearson, of London, who personally examined the whole particulars." There cannot, we think, be a doubt that their opinion was correct, and that nothing but the rapid termination prevented the development of the phenomena of fever in these cases; but in fairness to Dr. Christison, it should be added, that he considers "this opinion cannot be received with confidence by the medical jurist and the physician, since it is not supported by any previous account of the effects of sulphuretted hydrogen." Perhaps these cases may receive some confirmation from the following report (certainly not a full one) of a similar accident by Dr. Arnott.* "In a mews behind Bedford-square, a stable had been let for a time to a butcher, and a heap of dung had been formed at the door, containing pigs' offal, pigeons' dung, &c. During the act of

* Fourth Report of Poor Law Commissioners, p 106.

removing this heap, a coachman's wife and her three children, of an adjoining stable, sat for a time at an open window nearly over the place until the insufferable stench drove them away: two of the poor children died of the poison before 36 hours, and the mother and other child narrowly escaped."

In Dr. Christison's cases the description of the appearances of Peyer's glands exactly corresponds (especially the first case) with Dr. Bright's description, and with the representation given in one of the plates in his great work. As also with the minute investigations of Dr. Staberoh, who regards the first stage of the follicular affection, as "an infiltration into the mucous coat, and especially the crypts called Peyerian glands," but also taking place, as he has repeatedly seen it, in different parts of the colon, "and to which he considers the inflammation of the mucous membrane secondary."—(Dublin Journal, Vol. 13.)

But farther—if dothin-enterite were a consequence of disordered circulation depending upon an impression on the brain and spinal chord, we might expect to meet it in other cases in which these organs are engaged, as in the periodic fevers and in typhus: but we do not, nor can any other local inflammation be named as similarly constant in these diseases, and filling its place in the "febrile circle." On the contrary, M'Cartney, Armstrong, and others, have fully proved that the vascular congestion commonly found in these diseases is not of an inflammatory nature, and that, though it may remotely give rise in certain cases to an inflammatory re-action for its removal, it is yet a distinct pathological condition.*

Two other opinions may be entertained of the relation of dothin-enteritis to fever. One, that it is the primary cause: that fever is the sum of the symptoms of this inflammation—the other, that it is the specific effect of the septic poison from which typhoid fever originates, and like the other symptoms of this disease, merely a link in the chain of sequences constituting fever.

Perhaps the strongest arguments for the first of these opinions are the large proportion of cases of typhoid fever in which it is found to exist,—the influence it exercises upon the severity of the disease, and the effects of antiphlogistic remedies, more especially of topical blood-letting. It will be presently seen that the two first circumstances are equally well explained upon the second opinion. With reference to the effects of blood-letting it must be admitted that a very considerable amelioration of symptoms, and not unfrequently their total removal, has followed timely and free abstraction of blood, especially by leeches applied over the affected intestine, and not only are the tenderness and pain in the part, with the meteorism and diarrhœa thus relieved, but the head-ache, thirst, pulse, and other general symptoms commonly undergo at least a temporary and partial improvement. But it may be doubted whether this is to be attributed so much to the removal of inflammation as to an impression made upon the general disease, by the new movement given to the circulation in general by the smallest local abstraction of blood, and which is felt in every part of the system.

One or two circumstances may be cited in proof of this dynamic effect of bleeding, and to illustrate its application to the present case—1st, the well-known fact that the impression made upon the central organ of the circulation by the bleeding from a few leech-bites is totally disproportionate to the quantity taken away. 2nd. The effect of leeching, or bleeding in some other disorders in which no supposition of inflammation could exist. In amenorrhœa, for instance, we have known the disorder of several months' standing removed by the application of a few leeches to the inguinal region before the leeches were themselves removed. In ague also, an effect almost equally marked may be sometimes produced by the same means. The following short case illustrates this. C. F——, æt. 20, was admitted into the Navan Hospital on the 22nd of February, labour-

* For Dr. M'Cartney's observations, see Dub. Med. Trans. Vol. 2, p. 574.

ing under tertian ague. The fit comes on two hours earlier at each period. Has some tenderness on pressure, and fulness of epigastrium, thirst, tongue red at tip and edges—previous to entering hospital took two emetics and an aperient. A fit took place about 3 a. m. on the 21st, and might be expected to recur at one a. m. on the 23rd. I ordered 12 leeches to be applied on the evening of the 22nd to the epigastrium, and a draught to be taken at bed-time, containing 20 drops of laudanum. The fit occurred at 5 a. m. 4 hours later than it was expected. On the 25th it came on at 7, and the leeching being repeated on the evening of the 26th, he sweated copiously during the night, and had no return of the fit. There was a slight return of it five days after, but from this time he got rapidly well.

Without entering farther into the discussion of the theory, that dothinenteritis is *the cause* of the febrile phenomena, we pass on to submit certain considerations in support of the view which regards it as an effect not *of* the fever or of the state of the nervous and circulating systems produced by the fever, but as the direct effect of the poison itself—as one (probably the first) of the links in the chain of sequences, constituting fever, and one upon the occurrence of which some of the others may probably depend. This view approaches nearer to that of Louis, who is of opinion that the affection of the follicles occurs in the beginning of the disease, than to that of Chomel, who seems inclined to admit its classification among the secondary inflammations, but differs from the definition of the former eminent pathologist, inasmuch as it seeks to establish the agency of a morbid poison as *the cause of fever,* in place of his decision "that *the cause is unknown.*" The following extract from his summary of the diagnostic symptoms of typhoid fever is important, as containing two particulars which we shall find to have a bearing upon this inquiry.

"Maladie aiguë accompagnée d'un mouvement fébrile plus ou moins intense, variable dans sa durée; propre aux jeunes sujets, *principalement à ceux qui se trouvent depuis peu de temps au mileau de circonstances nouvelles pour eux,* dont la cause est inconnue; debutant par un frisson violent, l'anorexia, la soif, et dans la très grande majorité des cas *par des coliques et la diarrhée,*" &c.

The two circumstances here mentioned by Louis, of the subjects of typhoid fever being those newly exposed to influences, the nature of which he concludes are not known—and the diarrhœa which ushers in the complaint,—we conceive tend to support the theory that the intestinal affection is a consequence of the effort made by the excreting organs—more especially the liver—to rid the system of the poison which has been introduced into the blood. It is easy to conceive that the native of Paris, born and brought up in the atmosphere of its fosses, and drinking all his life the tainted waters of its wells and river, may habitually eliminate from the blood such products as are thus taken in unfit for assimilation or nutriment, and that to a constitution unused to such a task the consequence of taking into the circulation the same decomposing substances should be different. And, if we aid our conception of this fact by a reference to what takes place in the different classes of persons exposed to malaria of other kinds in temperate climates, we shall see why intestinal affections should be among the first consequences of the process; for it will appear that the liver is the organ by means of whose excretions the poison is attempted to be got rid of, and according to the facility with which this is performed or otherwise, will be the chances of escape or the contrary, from the effects of the poison. It is well known that, while the lungs of a native of a warm climate are liable to become diseased upon removal to a cold one, the liver is the organ prone to suffer upon the inhabitant of a cold removing to a warm climate. It is also found that the stranger from a colder country will rapidly contract fever from exposure to malaria in a temperate climate, while the person newly arrived from a warm one will not be similarly affected until that change in the order of his functions termed acclimatization has taken place, and he becomes assimilated in habits to the inhabitant of the same latitude.

*(To be concluded in our next.)*

